# Loss of Brain Angiogenesis Inhibitor-3 (BAI3) G-Protein Coupled Receptor in Mice Regulates Adaptive Thermogenesis by Enhancing Energy Expenditure

**DOI:** 10.3390/metabo13060711

**Published:** 2023-05-31

**Authors:** Haifa Alsharif, Mary N. Latimer, Katherine C. Perez, Justin Alexander, Md Mostafizur Rahman, Anil K. Challa, Jeong-A. Kim, Sasanka Ramanadham, Martin Young, Sushant Bhatnagar

**Affiliations:** 1 Department of Medicine, Division of Endocrinology, Diabetes, and Metabolism, Heersink School of Medicine, University of Alabama at Birmingham, Birmingham, AL 35294, USA; saba2323@uab.edu (H.A.); kperez24@uab.edu (K.C.P.); justinalexander@uabmc.edu (J.A.); mostafizurrahman@uabmc.edu (M.M.R.); jeongakim@uabmc.edu (J.-A.K.); 2 Comprehensive Diabetes Center, University of Alabama at Birmingham, Birmingham, AL 35294, USA; 3 Division of Cardiovascular Disease, Department of Medicine, Heersink School of Medicine, University of Alabama at Birmingham, Birmingham, AL 35294, USA; martinyoung@uabmc.edu (M.Y.);; 4 Department of Biology, University of Alabama at Birmingham, Birmingham, AL 35294, USA; akchalla@uab.edu; 5 Department of Cell, Developmental, and Integrative Biology, University of Alabama at Birmingham, Birmingham, AL 35294, USA

**Keywords:** BAI3, GPCR, energy expenditure, adaptive thermogenesis, body weight, brown adipose tissue, QMR, RER

## Abstract

Effective energy expenditure is critical for maintaining body weight (BW). However, underlying mechanisms contributing to increased BW remain unknown. We characterized the role of brain angiogenesis inhibitor-3 (BAI3/ADGRB3), an adhesion G-protein coupled receptor (aGPCR), in regulating BW. A CRISPR/Cas9 gene editing approach was utilized to generate a whole-body deletion of the *BAI3* gene (*BAI3^−/−^*). In both *BAI3^−/−^* male and female mice, a significant reduction in BW was observed compared to *BAI3^+/+^* control mice. Quantitative magnetic imaging analysis showed that lean and fat masses were reduced in male and female mice with BAI3 deficiency. Total activity, food intake, energy expenditure (EE), and respiratory exchange ratio (RER) were assessed in mice housed at room temperature using a Comprehensive Lab Animal Monitoring System (CLAMS). While no differences were observed in the activity between the two genotypes in male or female mice, energy expenditure was increased in both sexes with BAI3 deficiency. However, at thermoneutrality (30 °C), no differences in energy expenditure were observed between the two genotypes for either sex, suggesting a role for BAI3 in adaptive thermogenesis. Notably, in male *BAI3^−/−^* mice, food intake was reduced, and RER was increased, but these attributes remained unchanged in the female mice upon BAI3 loss. Gene expression analysis showed increased mRNA abundance of thermogenic genes *Ucp1*, *Pgc1α*, *Prdm16*, and *Elov3* in brown adipose tissue (BAT). These outcomes suggest that adaptive thermogenesis due to enhanced BAT activity contributes to increased energy expenditure and reduced BW with BAI3 deficiency. Additionally, sex-dependent differences were observed in food intake and RER. These studies identify BAI3 as a novel regulator of BW that can be potentially targeted to improve whole-body energy expenditure.

## 1. Introduction

Obesity results from a chronic imbalance between energy intake and expenditure [1,2]. It is a major risk factor for developing metabolic disorders such as cardiovascular disease, liver disease, and Type 2 diabetes (T2D) [3,4]. Of critical importance, adipose tissues regulate whole-body energy homeostasis [5,6]. In particular, white adipose tissue (WAT) is an endocrine organ that stores energy as triacylglycerols (TAGs) and regulates energy expenditure [7,8,9,10,11]. Its function is affected by the balance between de novo lipogenesis, catabolic lipolysis, and secretion of adipokines such as leptin, resistin, TNFα, and IL6. In a state of negative energy balance, WAT performs lipolysis and hydrolysis of TAGs, generating free fatty acids (FFAs) to meet energy demands [12,13]. Conversely, in a state of positive energy balance, TAGs are stored in WAT, resulting in fat accumulation [2,14]. Excess stored fat can lead to insulin resistance, obesity, and metabolic syndrome [2,15]. The brown adipose tissue (BAT) maintains energy expenditure (EE) using FFAs to generate non-shivering heat during cold exposure [16]. In healthy individuals and rodents, twenty percent of the EE is achieved by uncoupled respiration, attributed to BAT, which involves oxidizing FFAs in the mitochondria to generate heat without ATP [17,18]. With insulin resistance, obesity, and T2D, BAT activity is reduced, decreasing EE, which affects BW [19,20].

Sexual dimorphism exists in feeding behavior and whole-body energy parameters [21]. The total energy intake in males is higher than in females, even after correcting for the lean body mass and metabolic rate [22]. The high-fat feeding leads to higher weight gain in males vs. females [23,24]. Further, there exists a difference in the utilization of carbohydrates and lipids as fuel sources. In the post-absorptive state, males oxidize FFAs, whereas females utilize FFAs, storing them as TAGs. While during exercise, females use a more significant proportion of lipids relative to carbohydrates. In comparison, males preferentially use carbohydrates as a fuel source [25,26,27]. Healthy females have more adipose tissue mass, which contributes to enhanced FFA and intramyocellular lipid content, and have relatively less skeletal muscle than males. Yet, females are resistant to FFA-induced insulin resistance [28,29]. Concomitantly, impaired fasting glucose is more prevalent in males [30]. The sex-dependent differences that contribute to energy homeostasis remain elusive.

The brain angiogenesis inhibitor-3 (BAI3 or ADBRG3) is a member of the brain-specific angiogenesis inhibitor (BAI) subfamily of cell-adhesion G-protein coupled receptors (GPCRs) [31]. It comprises a large N-terminal extracellular region containing a complement C1r/C1s, Uegf, Bmp1 (CUB) domain, a hormone binding domain (HBD), four thrombospondin type 1 repeats (TSRs), a conserved GPCR autoproteolysis–inducing (GAIN) domain constituting an autoproteolysis site (GPS), 7-transmembrane regions, and an intracellular PDZ domain binding motif [31,32,33]. The intracellular C-terminal region of the BAI3 has been reported to couple with an inhibitory Gα_i_-protein to promote myoblast fusion, thereby regulating muscle fiber formation [34,35,36]. The GPCRs are implicated in regulating whole-body energy expenditure. These effects are attributed, in parts, to the Gα_s_-mediated activation of cyclic adenosine monophosphate (cAMP) signaling, which increases protein kinase A (PKA)-mediated lipolysis in BAT and WAT [37,38,39]. The subsequently released FFAs serve as substrates for thermogenesis [40]. Additionally, cAMP/PKA signaling phosphorylates proteins in the electron transport complex, increasing mitochondrial oxidation in WAT and *Ucp1* expression during cold-mediated thermogenesis [5,41,42,43]. Thus, enhanced cAMP signaling in WAT and BAT increases EE, thereby, improving insulin sensitivity and glucose homeostasis, and potentially BW loss. The physiological role of BAI3 in whole-body energy metabolism is unknown. Herein, we used a BAI3 gene-deletion mouse model combined with metabolic phenotyping to assess how BAI3 regulates whole-body energy metabolism in male and female mice.

## 2. Materials and Methods

### 2.1. Animal

All experiments involving live animals were carried out according to the protocols approved by the University of Alabama at Birmingham Institutional Animal Care and Use Committee (IACUC-21632). Animals were housed under pathogen-free conditions in individually ventilated cages at 23.2 °C to 23.9 °C and 37% to 41% relative humidity. The mouse housing facilities were maintained with a standard 12 h light–dark cycle (6 a.m.–6 p.m.). BWs were assessed weekly in these mice.

### 2.2. Generation of BAI3 Gene-Deletion Mice

CRISPR/Cas9 was used to delete exon 2 and exon 18 of the *BAI3* gene (*Adgrb3*) to generate a genetically modified C57BL6/J mouse. Single-cell embryos were microinjected with sgRNAs (were designed using (https://doi.org/10.1093/nar/gky354 (accessed on 29 May 2023)) and synthesized by in vitro transcription) (25 ng/mL of each, 50 ng/mL total) and Cas9 protein (40 ng/mL). F0 animals were subjected to PCR with the primer sequences below, indexed, and run on a MiSeq Nano 2 × 250 flow cell. Data were analyzed with CRISPResso (https://doi.org/10.1038/nbt.3583 (accessed on 29 May 2023)), and F0s carrying frameshift mutations were backcrossed to C57BL6/J mice. F1s were similarly characterized via MiSeq. The primer sequences are as follows: BAI3.ex2.F: acactctttccctacacgacgctcttccgatctNNNNNNTCAGATACGCCGGGTATTTCCAAC; BAI3.ex2.R: gtgactggagttcagacgtgtgctcttccgatctACATAAT TCCACAAGACTCGGTTCTCC; BAI3.ex18.F: acactctttccctacacgacgctcttccgatctNNNNN NCAGAGCATCTGCACGACCACCAC; and BAI3.ex18.R: gtgactggagttcagacgtgtgctcttcc gatctAGACACAAGTACCCTGGGAACAGTCAC. Consequently, the 4 bp deletion in exon 2 added a BslI site, while the 1 bp deletion in exon 18 added a TaqαI site. The sequence for the wild-type *BAI3* and *BAI3*-targeted alleles are as follows: Ex2.ref: TTGGTACTGAACAAGGTGAGCCCGAGCCAGTTTGGTTGCCATGTCTTATGCACTTGGTTGGAAA; Ex2.del: TTGGTACTGAACAAGGTGAGCCCGAGCTTGGTTGCCATGTCTTA TGCACTTGGTTGGAAA; Ex18.ref: CTTCATTCTGTTGGGTCTTGACAGAGGCGTGGCAGTCGTATATGGCTGTGACAGGAAAAATCAGGACCCGGCTT; and Ex18.del1: CTT CATTCTGTTGGGTCTTGACAGAGGCGTGGCAGTCGATATGGCTGTGACAGGAAAA ATCAGGACCCGGCTT.

### 2.3. Western Blotting

For protein extraction, 100 mg of frozen brain tissues were homogenized in 1 mL lysis buffer (20 mM Tris-HCl (pH 7.5), 150 mM NaCl, 1 mM Na_2_EDTA, 1 mM EGTA, 1% Triton, 2.5 mM sodium pyrophosphate, 1 mM β-glycerophosphate, 1 mM Na_3_VO_4_, and protease inhibitor cocktail). Samples were homogenized, sonicated for 10 s, and subjected to high-speed spin at 13,000 rpm for 10 min at 4 °C. The supernatant was collected, and protein concentration was determined via BCA assay. Protein samples prepared by adding 10 mM DTT and 20 mM β-mercaptoethanol were subjected to SDS-PAGE electrophoresis using 8.5% polyacrylamide gel. The anti-mouse BAI3 (1:3000) antibody was used to determine the relative protein abundance of BAI3. The polyclonal anti-mouse BAI3 antibody was custom-generated using a C-terminal BAI3 protein fragment to immunize rabbits (as described [44]) (GRREVQDAFRCRLRNCQDPINADSSSSFPNGHAQIMTDFEKDVDIACRSVLHKDIPCRAATITGTLSRISLNDDEEEKGTNPEGLSYSTLPGNVISKVIIQQPTGLH MPMSMNELSNPCLKKENTELRRTVYLCTDDNLRGADMDIVHPQERMMESDYIVMP- RSSVSTQPSMKEESKMNIGMETLPHERLLHYKVNPE). After immunization, the anti-sera were collected, and the anti-BAI3 antibody was purified. Secondary antibodies used were goat anti-mouse (1:5000) and donkey anti-rabbit (1:5000). Elov3 anti-goat (sc-54879) and Dio2 anti-rabbit (sc-98716) antibodies were from Santa Cruz Biotechnology.

### 2.4. Primer Design and PCR

The forward 1 (F1) primer (5′-TGAACAAGGTGAGCCCGAGCCAGT-3′) and reverse (R) primer 5′-AGGCAACCCTCCGTCTGCTCATT-3′ were designed to amplify the wild-type BAI3 allele. Whereas the F2 primer (5′-TGAACAAGGTGAGCCCGAGCTTGG-3′), lacking the four bp nonsense mutations generated due to CRISPR/Cas9 targeting, and RP, were used to amplify cDNA from the *BAI3^−/−^* mouse tissues.

### 2.5. Quantitative Magnetic Resonance (QMR)

The mouse body composition (fat and lean mass) was determined using an EchoMRI™ 3-in-1 QMR machine (Echo Medical Systems, Houston, TX, USA). A system test was performed using a known fat standard before the measurements. Mice were weighed and placed into a clear holding tube capped with a stopper that restricted vertical movement but allowed constant airflow. The tube was inserted into the machine, and the mouse was scanned using a primary accumulation of 1 for measuring total body water in addition to fat and lean mass. The QMR measurements were done in the UAB Small Animal Phenotyping Core supported by the NIH Nutrition & Obesity Research Center P30DK056336, Diabetes Research Center P30DK079626, and the UAB Nathan Shock Center P30AG050886A according to their standard protocols.

### 2.6. Cold Exposure, Comprehensive Laboratory Animal Monitoring System, and Thermoneutrality

Eight-week-old wild-type mice (on C57BL6/J background) were housed at the Center for Comparative Medicine at the University of Alabama at Birmingham under controlled conditions (25 ± 1 °C and 12 h:12 h light–dark cycle enforced by environmental chambers) within a Comprehensive Laboratory Animal Monitoring System (CLAMS; Columbus Instruments Inc., Columbus, OH, USA). All mice were housed singly and allowed to acclimatize to the CLAMS for 9 days before initiation of the experimental protocol. During the acclimatization period, mice received a standard rodent chow diet and had access to water. Subsequently, in the indirect-calorimetry chambers, the oxygen consumption and carbon dioxide production were measured once every 9 min. The total energy expenditure represented the average hourly energy expenditure over 72 h. Resting energy expenditure was calculated from the average over three nonconsecutive 10 min intervals during which energy expenditure was minimal, with at least 1 h between each period. Infrared beams determined locomotor activity for horizontal (x, y) activity. The standard chow diet provided in all studies was obtained from the Harlan NIH-31 Irradiated Open Formula Mouse/Rat Diet (4.7% fat). Time-of-day-dependent analysis for food intake, RER, EE, and activity exhibiting significant oscillations was performed using Cosinor analysis (as described [45,46]). Briefly, Cosinor analyses were conducted using nonlinear regression with PRISM GraphPad (Version 9.4.1(458)) and by fitting the data on the cosine curve, assuming that the free-running period is fixed at 24 h. Data were considered rhythmic if the *p*-value of the R2 of the linearized Cosinor function of f(t) = M + Cos(2πt/24) + Sin(2πt/24) was less than 0.05. In cases where both comparison groups significantly fit the cosine function, individual parameter estimates for mesor, amplitude, and phase were compared using t-tests determined from the pooled variances. The null hypothesis of no model effects was rejected in all analyses at *p* < 0.05.

### 2.7. Tissue Histology and Analysis

Freshly extracted adipose tissues were fixed with 4% paraformaldehyde, dehydrated, embedded in paraffin, and sectioned. Sections were stained with hematoxylin (H) and eosin (E). Images were processed for cell size using the Olympus cell Sens Imaging Software version 1.18 (RRID: SCR_014551). The average area of the eWAT lipid droplet was assessed through a semi-automated method.

### 2.8. Quantitative Real-Time PCR

Total RNA was isolated from tissues using TRIzol™ reagent (Invitrogen, cat# 15596018). Following RNA extraction, cDNA was synthesized using a high-capacity cDNA reverse transcription Kit (Applied Biosystems, cat# 4368814). The mRNA abundance of genes was determined by qPCR (ABI3000, Applied Biosystems) with PCR using Fast Start SYBR Green (Roche, cat# 4673484001). The steady-state mRNA levels for each gene were calculated via the comparative ΔCT method and expressed relative to β-actin mRNA. Primer sequences used for qPCR are listed in Table 1.

### 2.9. Statistical Analysis

Data are represented as means ± SEM. Statistical significance was performed using Student’s two-tailed unpaired t-test for independent data. The significance limit was set at *p* < 0.05. Statistical analysis was performed using two-way or repeated-measure ANOVA. PRISM GraphPad (Version 9.4.1(458)) was used to perform a two-way ANOVA to investigate the main effects due to genotype. Repeated-measure ANOVA was used to determine the impact of genotype on energy homeostasis. A full model, including second-order interactions, was conducted for each experiment. Significant differences were determined using Type III sums of squares. The null hypothesis of no model effects was rejected at *p* < 0.05. Rhythmic parameter comparisons (phase and amplitude) of CLAMs data were calculated using Cosinor analysis. For the following equation, the initial values and default parameter ranges were set manually: f(t) = Mesor + A × Cos [(2 πt/T) + Acrophase]; where mesor (an acronym for midline estimating statistic of rhythm) = the mean of the oscillation; A = the amplitude (peak-to-trough difference); T = the period (24 h); acrophase = the timing of the cosine maximum; t = a timepoint.

## 3. Results

### 3.1. Generation and Validation of BAI3^−/−^ Mice

**Figure 1:**
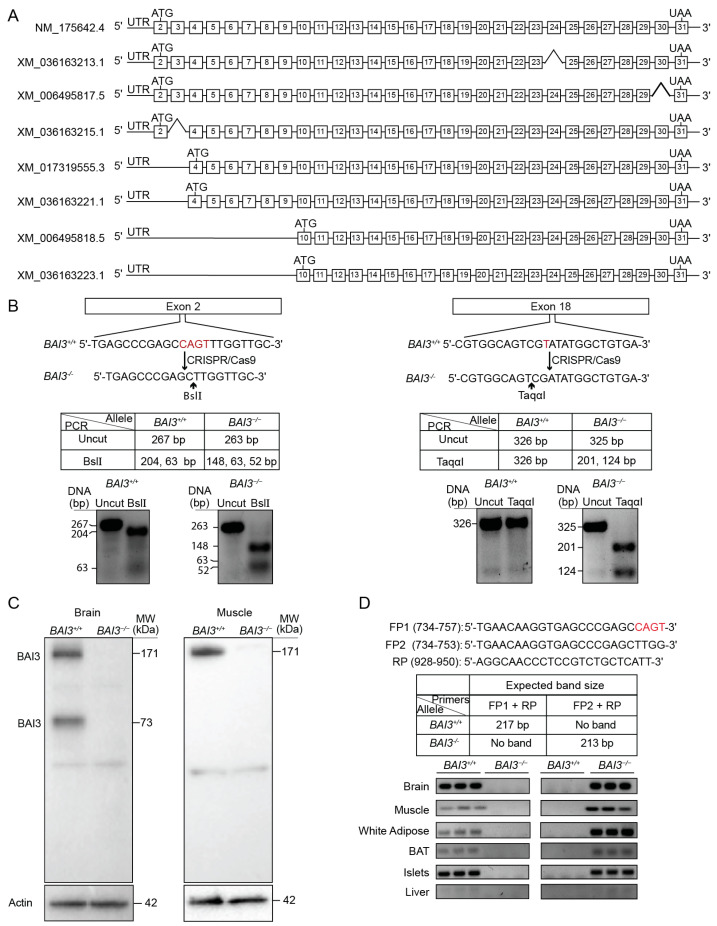
BAI3-deletion mice were generated using CRISPR/Cas9. (**A**) Coding exons for the full-length and seven predicted BAI3 transcripts. (**B**) CRISPR/Cas9 targeted *BAI3^+/+^* exon 2 and exon 18 for generating *BAI3^−/−^* mice. Genotyping by PCR to show mutations in exon 2 and exon 18 of BAI3. (**C**) Western blots show protein expression in the whole brain and muscle lysates isolated from *BAI3^+/+^* and *BAI3^−/−^* mice (n = 4). Actin was used as a loading control. (**D**) The tissue expression profile of BAI3 was determined by PCR using primers designed to differentiate between the cDNA obtained from *BAI3^+/+^* and *BAI3^−/−^* mice. Actin was used as a loading control to show the presence of DNA (n = 3).

The BAI3-deletion (*BAI3^−^*^/*−*^) mice on a C57BL/6J background were generated using CRISPR/Cas9 genome editing technology [47]. The full-length BAI3 transcript (NCBI ID: NM_175642.4) contains 30 coding exons, from exon 2 to 31 (Figure 1A), that encodes an approximately 171 kDa size protein. The NCBI database lists seven additional predicted isoforms of BAI3. XM_036163213.1, XM_006495817.5, and XM_036163215.1 isoforms lack exon 24, exon 30, and exon 3, respectively, from the coding region spanning from exon 2 to exon 31, plausibly due to splicing. XM_017319555.3 and XM_036163221.1 isoforms are predicted to encode identical proteins translated from exon 4 to exon 31. Isoforms XM_006495818.5 and XM_036163223.1 are predicted to encode the shortest BAI3 protein fragment from exon 10 to exon 31 (Figure 1A). To achieve complete deletion of the BAI3 from mice, exon 2 and exon 18 were targeted by CRISPR/Cas9. The F1 mice produced from the breeding of the founder mouse carried a 4 bp deletion (CAGT) in exon 2 and a 1 bp deletion (T) in exon 18, generating a premature stop codon and stopping protein translation. Further, these nonsense mutations introduced BslI and TaqαI restriction enzyme digest sites in exon 2 and exon 18, respectively. Mice carrying a mutation in exon 2 and exon 18 on the same allele were selected for breeding. Genotyping was performed by polymerase chain reaction (PCR) followed by restriction enzyme digest (Figure 1B) using tail DNA from the wild-type control and BAI3-mutant mice. The PCR-amplified regions of exon 2 and exon 18 were subjected to BslI and TaqαI restriction enzyme digest, respectively, followed by DNA gel electrophoresis. As predicted, BslI digestion of the PCR-amplified region of exon 2 generated 204 and 63 bp DNA fragments from the *BAI3^+^*^/*+*^ allele, whereas 148, 63, and 52 bp DNA fragments were generated from the BAI3-targeted allele. Digestion by TaqαI of the PCR-amplified exon 18 generated a 326 bp DNA fragment from the *BAI3^+^*^/*+*^ allele, whereas 201 and 124 bp DNA fragments were generated from the BAI3-mutated allele. The absence of 204 and 326 bp DNA fragments after BslI and TaqαI digestion confirmed homozygous mutation on the same allele in mice in exon 2 and exon 18 of the BAI3 gene (Figure 1B). Western blot analysis demonstrated the presence of the full-length BAI3 protein (~171 kDa; theoretical molecular weight = 171.13 kDa) in brain and muscle lysates prepared from *BAI3^+^*^/*+*^ mice. The complete absence of this band in the brain and muscle of mice containing the BAI3-mutated allele confirmed the presence of BAI3-deletion mice (*BAI3^−^*^/*−*^) (Figure 1C). In the brain, we also observed a ~73 kDa in control but not in *BAI3^−^*^/*−*^ mice. It is possible that this ~73 kDa band observed in the brain corresponds to a protein fragment generated from BAI3 isoforms, and it has been reported previously [33].

### 3.2. Tissue Expression Profile of BAI3

We assessed the tissue expression profile of BAI3. PCR was performed using cDNA from tissues of *BAI3^+/+^* and *BAI3^−/−^* mice. Primers were designed to differentiate between *BAI3^+/+^* and BAI3-CRISPR-targeted alleles (Figure 1D). The forward (F1, containing CAGT sequence present in the exon 2 of *BAI3^+/+^* mice) and reverse (R) primers were designed to generate a 217 bp DNA fragment when using cDNA prepared from *BAI3^+/+^* mouse tissues, but no band was expected from *BAI3^−/−^* mouse tissue cDNA. Alternatively, FP2 (lacking CRISPR-targeted CAGT bases) and RP were designed to generate a 213 bp DNA fragment from cDNA prepared from tissues obtained from *BAI3^−/−^* but not from *BAI3^+/+^* mice. Tissues such as brain, muscle, WAT, BAT, and islets from the *BAI3^+/+^* mice expressed only the 217 bp band (Figure 1D). In contrast, *BAI3^−/−^* mouse tissues expressed only the 213 bp fragment. These findings confirm the deletion of BAI3 in the *BAI3^−/−^* mice. No or very low expression of BAI3 was detected in the liver (as was reported previously [48]). A similar tissue expression profile of BAI3 was observed using quantitative PCR (Figure S1).

### 3.3. BAI3^−/−^ Mice Have Reduced Body Weight

**Figure 2:**
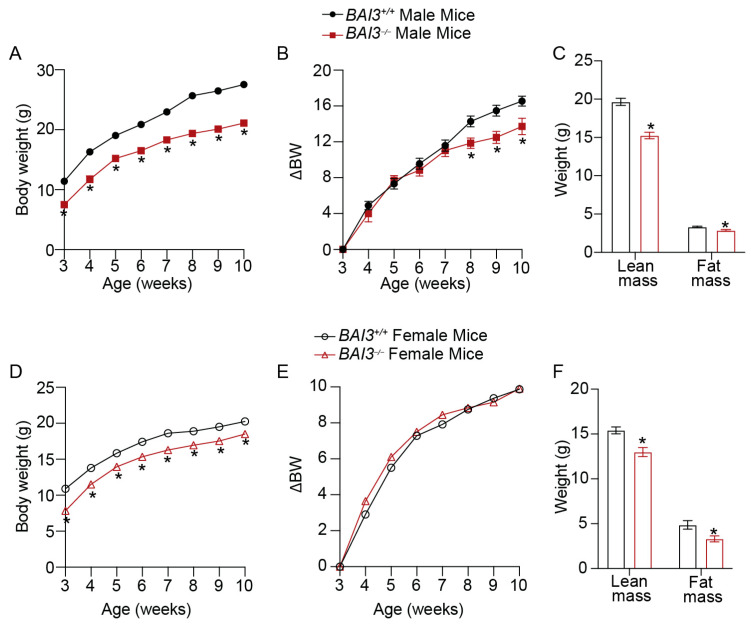
BW measurements in male and female mice. (**A**) Weekly BW of male *BAI3^−/−^* and *BAI3^+/+^* mice (n = 17–20/genotype). (**B**) BWs of male mice after the baseline correction (expressed relative to the BW at three weeks) (n = 17–20/genotype). (**C**) Body composition (fat and lean masses) of male *BAI3^−/−^* and *BAI3^+/+^* (n = 15/genotype) mice. (**D**) Weekly BW of female *BAI3^−/−^* and *BAI3^+/+^* mice (n = 15–20/group). (**E**) BWs of female mice after the baseline correction (expressed relative to the BW at three weeks) (n = 15–20/genotype). (**F**) Body composition (fat and lean mass) of female *BAI3^−/−^* and *BAI3^+/+^* mice (n = 5/genotype). Data are expressed as mean ± SEMs, * *p* < 0.05.

The BWs of male and female *BAI3^+/+^* and *BAI3^−/−^* mice on a standard chow diet were measured weekly. A significant reduction in the BW was observed at 3 weeks of age in both male (ΔBW = 3.6 g, 32% decrease, *p* < 0.01; ΔBW is the difference between the average BW of *BAI3^−/−^* and *BAI3^+/+^* mice) and female (ΔBW = 3.0 g, 27% decrease, *p* < 0.01) *BAI3^−/−^* mice compared to the *BAI3^+/+^* mice (Figure 2A,D). Furthermore, the reduction in BW between genotypes in male (ΔBW = 6.4 g, 23.35% decrease, *p* < 0.01) and female (ΔBW = 1.75 g, 8.6% decrease, *p* < 0.01) mice was sustained until 10 weeks of age (Figure 2A,D). After baseline correction (BW of 3-week-old *BAI3^+/+^* and *BAI3^−/−^* mice were set to 0), further decreases in the BWs of male (ΔBW at 10 weeks = 2.81 g, 16.9% decrease, *p* = 0.02) but not female (ΔBW at 10 weeks = −0.025 g) *BAI3^−/−^* mice were observed compared to *BAI3^+/+^* mice (Figure 2B,E). This suggests that BAI3 deletion differentially affects BW in adult (after 6–7 weeks of age) males and females. Next, we determined lean and fat masses in 8-week-old mice by QMR. In both male and female mice, significant reductions in lean mass (male = 22% decrease, *p* < 0.01; female = 15.6% decrease, *p* = 0.01) and fat mass (male = 13% decrease, *p* < 0.01; female = 32% decrease, *p* = 0.02) were observed in the *BAI3^−/−^* mice compared to the *BAI3^+/+^* mice (Figure 2C,D). However, no difference between the genotypes was observed after normalizing lean or fat mass with the total BW (Figure S2). These findings reveal that BAI3 regulates BW in both males and females. Moreover, the deletion of BAI3 has an additional effect on the BW in adult male mice.

### 3.4. Loss of BAI3 Decreases WAT Tissue Weight

**Figure 3:**
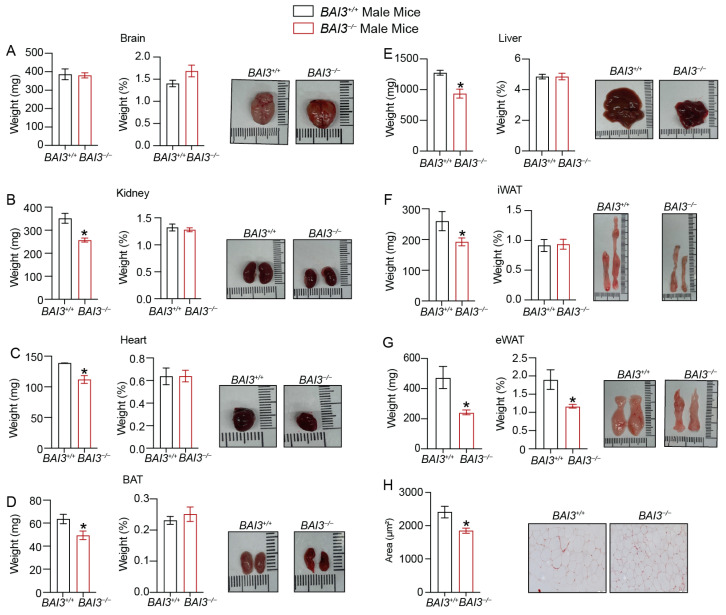
Assessment of weight of tissues obtained from *BAI3^+/+^* and *BAI3^−/−^* male mice. (**A**–**G**) Tissues were harvested from eight-week-old *BAI3^+/+^* and *BAI3^−/−^* male mice. The absolute tissue weight (**left**), normalized tissue weight to the total BW (**middle**), and gross morphology (**right**) for the brain, liver, kidney, heart, eWAT, iWAT, and BAT are presented (n = 8–10). (**H**) H and E staining of eWAT for determining adipocyte size. Data are expressed as mean ± SEMs, * *p* < 0.05.

**Figure 4:**
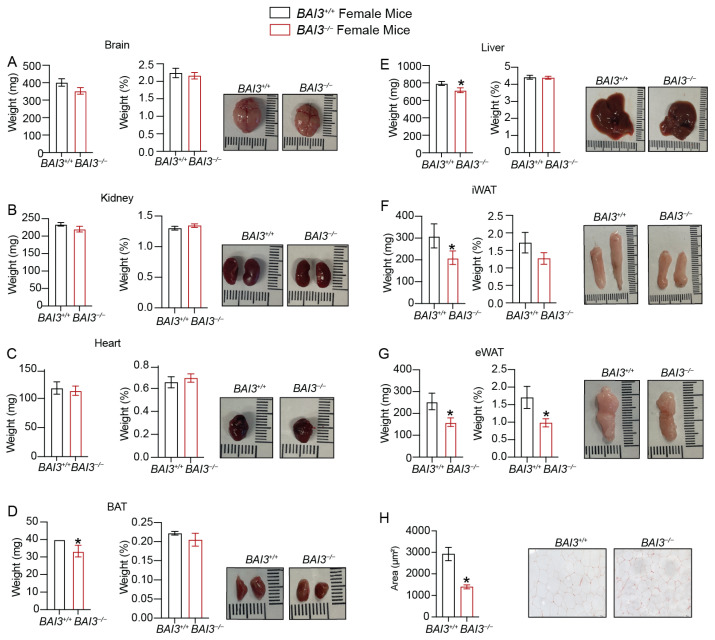
Assessment of weight of tissues obtained from *BAI3^+/+^* and *BAI3^−/−^* female mice. (**A**–**G**) Tissues were harvested from eight-week-old *BAI3^+/+^* and *BAI3^−/−^* female mice. Absolute tissue weight (**left**), normalized tissue weight to the total BW (**middle**), and gross morphology (**right**) for the brain, liver, kidney, heart, eWAT, iWAT, and BAT are presented (n = 6–8). (**H**) H and E staining of eWAT for determining adipocyte size. Data are expressed as mean ± SEMs, * *p* < 0.05.

The reductions in BW are often associated with decreases in WAT tissue weight and adipocyte size/number, which improves inflammatory characteristics and aids in the clearance of stored energy [14,49]. To determine if the loss of BAI3 regulates adipose tissue weight, we assessed the weight of tissues harvested from male and female *BAI3^−/−^* and *BAI3^+/+^* mice. In male mice, significant reductions in kidney, heart, and liver weights (Figure 3B,C,E), but not of the brain (Figure 3A), were noted in the *BAI3^−/−^* mice compared to that in the *BAI3^+/+^* control mice. However, normalization to BW revealed no significant differences in tissue weights between genotypes. Among the fat compartments, BAT, inguinal white adipose tissue (iWAT), and epididymal white adipose tissue (eWAT) weights were decreased in the *BAI3^−/−^* mice when compared to that in the *BAI3^+/+^* control mice (Figure 3D,F,G). Normalization with the BW revealed a significant lowering of eWAT weight (~30% decrease; *p* = 0.02), but not of iWAT and BAT, in the *BAI3^−/−^* mice compared to that in the *BAI3^+/+^* control mice. Moreover, the size of adipocytes in the eWAT was significantly decreased in BAI3-deficient mice (Figure 3H). In female mice, brain, kidney, heart, and liver weights were similar (Figure 4A–C,E) in the *BAI3^−/−^* mice compared to the control *BAI3^+/+^* mice. As in males, normalization to the BW did not reveal any significant differences in the tissue weights between the genotypes in females. The eWAT, iWAT, and BAT tissue weights were significantly reduced in the *BAI3^−/−^* mice compared to the control *BAI3^+/+^* mice (Figure 4D,F,G). After normalization to BW, only eWAT tissue weight was significantly lowered (42% decrease, *p* = 0.04) (Figure 4G) along with the reduction in adipocyte size (Figure 4H) in the *BAI3^−/−^* mice compared to the control *BAI3^+/+^* mice. These outcomes suggest that the loss of BAI3 decreases eWAT tissue weight as a percent of total BW and size of the adipocytes in both male and female mice.

### 3.5. BAI3^−/−^ Mice Have Enhanced Energy Expenditure

**Figure 5:**
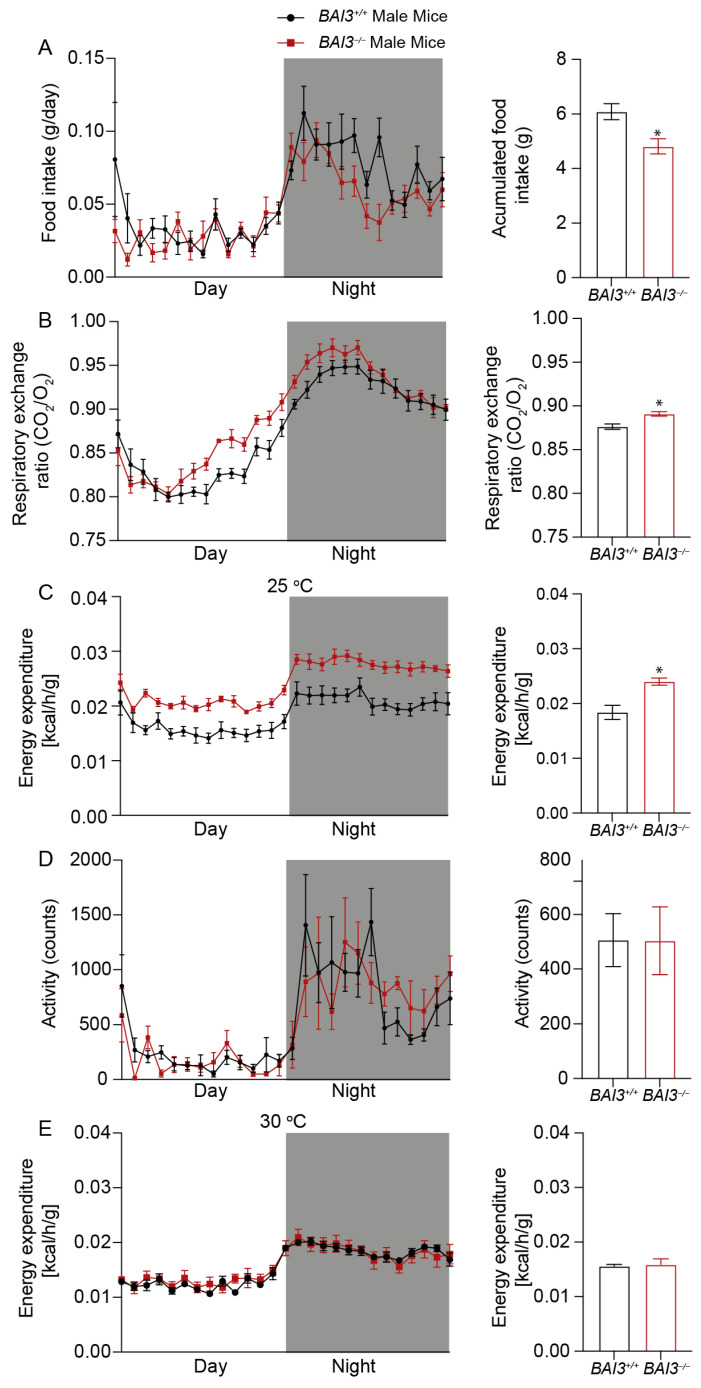
Measurement of whole-body energy parameters in *BAI3^+/+^* and *BAI3^−/−^* male mice. Food intake (**A**), respiratory exchange ratio (**B**), energy expenditure at 25 °C (**C**), total activity (**D**), and energy expenditure at 30 °C (**E**) in 8-week-old mice. The black and white bars in the left panels represent dark and light cycles over 24 h. Statistical analysis was performed using a two-way ANOVA with repeated measures (n = 6). Right panels, the quantitation of the data presented in the left panels averaged over a 24 h period (night and day combined) expressed as mean ± SEMs, * *p* < 0.05.

**Figure 6:**
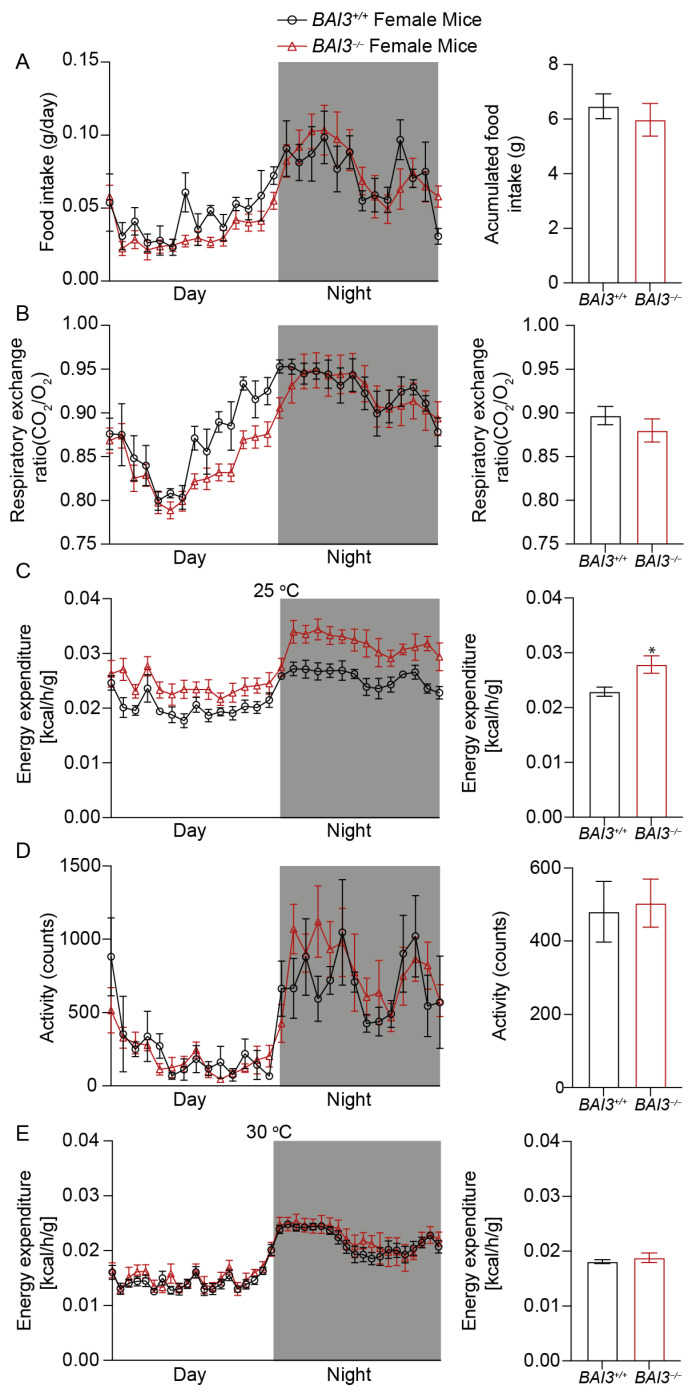
Measurement of whole-body energy parameters in *BAI3^+/+^* and *BAI3^−/−^* female mice. Food intake (**A**), respiratory exchange ratio (**B**), energy expenditure at 25 °C (**C**), total activity (**D**), and energy expenditure at 30 °C (**E**) in 8-week-old mice. The black and white bars in the left panels represent dark and light cycles over 24 h. Statistical analysis was performed using two-way ANOVA with repeated measures (n = 6). Right panels, the quantitation of the data presented in the left panels averaged over a 24 h period (night and day combined) expressed as mean ± SEMs, * *p* < 0.05.

To gain insights into factors that caused reductions in BW upon the loss of BAI3, whole-body energy balance parameters were assessed at eight weeks of age in male and female *BAI3^−/−^* and *BAI3^+/+^* mice housed at room temperature (~25 °C), a temperature that is below thermoneutrality for mice. More specifically, mice were housed in CLAMS cages (at ~25 °C) to determine whether BAI3 deletion impacted either behavior (i.e., physical activity, food intake) or metabolism (i.e., EE, RER). We initially performed repeated measures, two-way ANOVA, to investigate the main effects of genotype and time-of-day. Independent of the time-of-day, male *BAI3^−/−^* mice exhibited a significant decrease in food intake (20.9%, *p* = 0.01) (Figure 5A) and concomitant increases in RER (*p* < 0.01) (Figure 5B) and energy expenditure (23.3%, *p* = 0.01) (Figure 5C) relative to *BAI3^+/+^* mice. In contrast, no difference in physical activity was observed due to genotypes in male mice (Figure 5D). In female *BAI3^−/−^* mice, a significant increase in energy expenditure (17.74%, *p* = 0.04) was observed (in the absence of genotype main effects for food intake, physical activity, and RER) (Figure 6A–D). Given that all energy balance parameters exhibited significant time-of-day main effects in male and female mice, we next performed Cosinor analysis. In all instances, energy balance parameters significantly fit cosine curves with a periodicity of 24 h, allowing for the comparison of mesor, amplitude, and phase between *BAI3^−/−^* and *BAI3^+/+^* mice (Table 2 and Table 3). In male mice, mesor was significantly altered for all four energy balance parameters; RER (0.883 ± 0.002 vs. 0.876 ± 0.001; *p* < 0.01) and energy expenditure (0.0225 ± 0.00016 vs. 0.0183 ± 0.00015; *p* = 6.45 × 10^−19^) were increased, while food intake (0.043344 ± 0.0014 vs. 0.054785 ± 0.002; *p* = 7.57 × 10^−5^) and physical activity (460.992 ± 19.14 vs. 527.513 ± 24.02; *p* = 0.04) were decreased in male *BAI3^−/−^* mice. In addition, male *BAI3^−/−^* mice exhibited an increase in the RER amplitude (0.083 ± 0.002 vs. 0.072 ± 0.002; *p* < 0.01), while food intake was phase-advanced by ~1 h (16.17 ± 0.27 vs. 17.22 ± 0.33; *p* = 0.02). In female *BAI3^−/−^* mice, RER was significantly altered at the levels of mesor (decreased) (0.88 ± 0.002 vs. 0.897 ± 0.002, *p* = 1.05 × 10^−6^), amplitude (increased) (0.068 ± 0.002 vs. 0.056 ± 0.003, *p* < 0.01), and phase (~1.3 h phase delay; 16.83 ± 0.13 vs. 15.52 ± 0.17, *p* = 4.4 × 10^−7^). Significant increases were also observed in female *BAI3^−/−^* mice for energy expenditure mesor (0.0262 ± 0.00017 vs. 0.02 ± 0.00015, *p* = 2.65 × 10^−15^) and food intake amplitude (0.032829 ± 0.0019 vs. 0.025098 ± 0.0031; *p* = 0.03). Collectively, these data highlight select sex-specific differences in food intake and RER in *BAI3^−/−^* mice and increased energy expenditure independent of sex.

We next investigated the potential mechanisms by which energy expenditure was increased in male and female *BAI3^−/−^* mice. We hypothesized that increases in energy expenditure in *BAI3^−/−^* mice were secondary to the augmentation of thermogenesis. To initially test this hypothesis, energy expenditure was investigated in *BAI3^−/−^* and control mice acclimatized to thermoneutrality. More specifically, male and female *BAI3^−/−^* and control mice were placed in CLAMS cages at (~30 °C) for 10 days; during the last 2 days, energy expenditure was assessed. Under thermoneutrality conditions, neither a two-way ANOVA nor Cosinor analysis revealed significant differences in energy expenditure between male and female *BAI3^−/−^* mice (relative to controls) (Figure 5E and Figure 6E). Altogether, these data suggest a role for BAI3 in thermogenesis.

### 3.6. BAI3^−/−^ Mice Exhibit Increased Expression of Genes Involved in Thermogenesis

**Figure 7:**
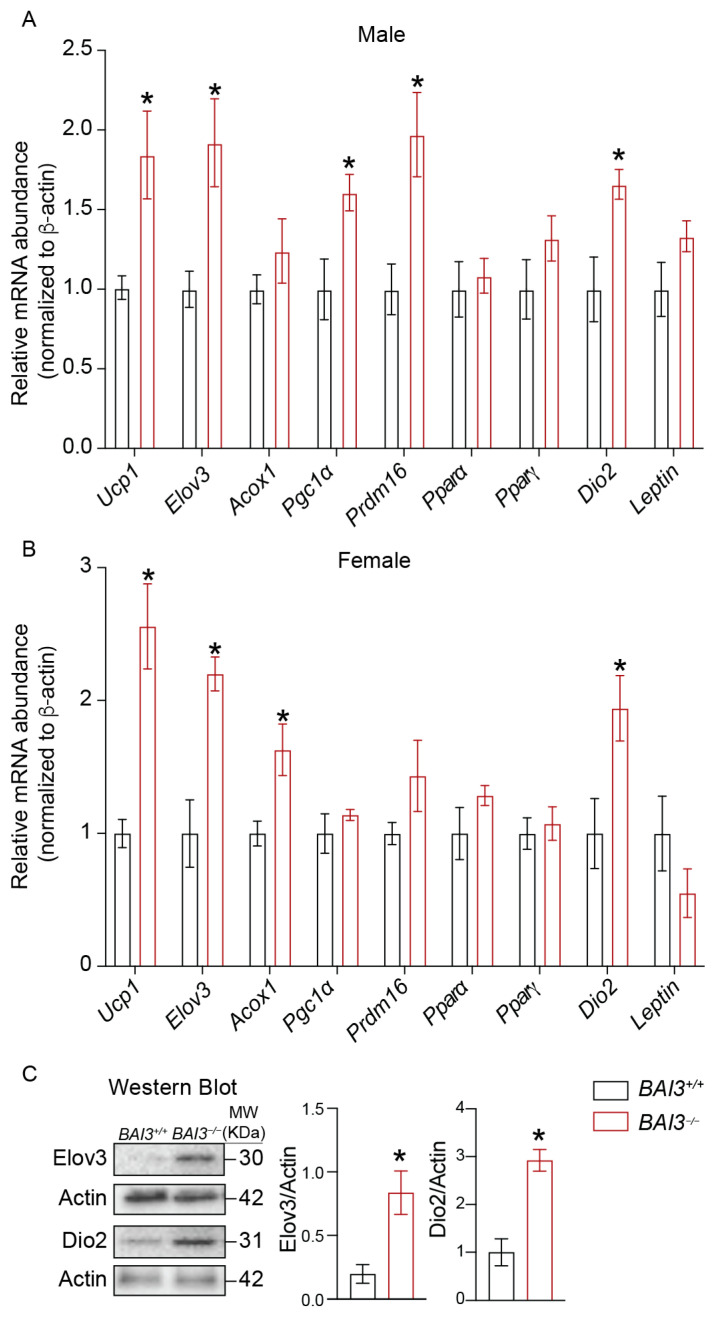
The mRNA and protein abundance in BAT. Total mRNA was prepared from BAT. The steady-state mRNA abundance of genes was expressed relative to the β-actin was determined using qPCR in males (**A**) and females (**B**). Western blots assessing the relative protein abundance normalized to actin (**C**). Data are reported as the mean ± SEMs, n = 4, * *p* < 0.05.

Analyses of the CLAMS data showed that the loss of BAI3 increases EE for maintaining adaptive thermogenesis (Figure 5 and Figure 6). Since BAT is a key tissue involved in energy expenditure during adaptive thermogenesis, by quantitative PCR, the steady-state mRNA abundance of thermogenic genes in BAT harvested from 8-week-old male and female *BAI3^−/−^* and *BAI3^+/+^* mice was determined to gain insights into how the loss of BAI3 regulates thermogenesis. These analyses revealed increased expression of peroxisome proliferator-activator receptor γ-coactivator 1α (*Pgc1*α) (60%, *p* < 0.05), uncoupling protein-1 (*Ucp1*) (75.28%, *p* < 0.05)*,* fatty acid elongase 3 *(Elov3)* (92%, *p* < 0.05), PR/SET domain 16 (*Prdm16)* (97%, *p* < 0.05)*,* and iodothyronine deiodinase 2 (*Dio2)* (66%, *p* < 0.05) in BAT obtained from the male *BAI3^−/−^* mice compared to that in the *BAI3^+/+^* mice (Figure 7A). Consistently, significant increases in the expression of *Ucp1* (2.5-fold, *p* < 0.05), *Elov3* (2.1-fold, *p* < 0.05), acyl CoA oxidase 1 (*Acox 1*) (50%, *p* < 0.05), and *Dio2* (two-fold, *p* < 0.05) were also observed in BAT tissue obtained from BAI3-deficient female mice. Moreover, the expression of *Ppar*α, *Pparγ*, and *Leptin* remained unchanged. Subsequently, protein quantification was performed using BAT tissue obtained from 8-week-old male *BAI3^−/−^* mice and *BAI3^+/+^* mice. Approximately a three-fold (*p* < 0.05) increase in the protein levels of Elov3 and Dio2 was observed in BAT (Figure 7C) with BAI3 loss. Increased expression of genes (*Ucp1*, *Elov3*, *Prdm16*, *Dio2*, and *Pgc1*α) and proteins (Elov3 and Dio2) involved in thermogenesis suggests that the loss of BAI3 increases BAT activity, providing insights into how BAI3 deletion increases EE.

## 4. Discussion

BAI3 is expressed in the central nervous system, hippocampal neurons, Purkinje cells, heart, islets, testis, muscles, and small intestine [35,49,50,51,52,53]. BAI3 is involved in diverse cellular functions, such as dendrite morphogenesis and synaptogenesis in Purkinje cells [54,55]. Further, BAI3 mediates synaptic connections between the anterior olfactory nucleus (AON) and granule cells in the olfactory bulb (OB), which is essential for the social transmission of food preference (STFP) [56]. In muscle, BAI3 interaction with the plasma membrane receptor protein stablin-2 was reported to regulate muscle myoblast fusion, a critical process determining muscle size and regeneration in adults [34,35,57]. In pancreatic islets, BAI3 is implicated in regulating insulin secretion [52]. These studies show that BAI3 is expressed in key metabolic tissues. However, its role in whole-body energy homeostasis remains unknown.

This study reports findings from the initial *BAI3^−/−^* mice characterization. These mice were generated by targeting exon 2 and exon 18 of the BAI3 gene by CRISPR/Cas9 approach to get a complete loss of protein translation from BAI3 transcripts (Figure 1). The deletion of full-length BAI3 protein was confirmed by the custom-made rabbit anti-mouse BAI3 antibody, which was generated in response to the epitope located in the intracellular C-terminal region of the BAI3 protein, common to all putative transcripts.

Our studies reveal (a) that the loss of BAI3 from both male and female mice leads to reduced BW, which is attributed to decreased food intake and enhanced energy expenditure, (b) reduced eWAT tissue weight and increased expression of genes and proteins known to enhance BAT activity, and (c) no differences in energy expenditure between *BAI3^+/+^* and *BAI3^−/−^* mice at thermoneutrality. These data suggest that BAI3 regulates food intake and maintains adaptive thermogenesis by enhancing energy expenditure to regulate BW in adult mice.

cAMP signaling is critical to the functioning of WAT and BAT in regulating whole-body energy homeostasis. In both WAT and BAT, increases in cAMP signaling in response to the activation of Gα_s_-coupled GPCRs such as β3-adrenoreceptor (AR) enhance PKA-mediated lipolysis to break down stored triacylglycerols to free fatty acids, which are an energy source in peripheral tissues [37,38,39]. Further, the activation of cAMP/PKA signaling in BAT increases the expression of *Ucp1,* a key thermogenic gene that increases cold-induced thermogenesis (adaptive thermogenesis), enhancing whole-body energy expenditure and insulin sensitivity [5,41,42,43]. Our data show that BAI3 is expressed in WAT and BAT. Furthermore, *BAI3^−/−^* mice exhibited reduced WAT tissue weight (in males and females), increased expression of thermogenic genes in BAT of males and females, and enhanced energy expenditure in the chronic cold. Thus, it is possible that the loss of BAI3, which is known to be coupled to the Gα_i_ [34], could lead to increases in cAMP signaling-mediated energy expenditure. In muscle, BAI3 was reported to be coupled with Gα_i_ [34]. Similarly, the BAI3 N-terminal fragment that binds its ligand complement 1q-like 3 (C1ql3) secreted protein was reported to effectively block the inhibitory effects of C1ql3 on insulin secretion from mouse islets in response to cAMP stimulation [52]. The molecular mechanism by which BAI3 regulates cAMP signaling and the functioning of adipose tissues remains to be elucidated.

Our study revealed sex-dependent differences in mice after the deletion of BAI3. Overall greater differences in the BW due to BAI3 deletion were observed in the male mice compared to the female mice. After baseline correction by normalizing BW for both genotypes at 3 weeks of age to zero, the male but not the female *BAI3^−/−^* mice exhibited a significant reduction in BW from 8 to 10 weeks of age, suggesting that the loss of BAI3 decreased BW only in the adult male mice. This reduction in the BW may remain sustained after 10 weeks of age. The sexual dimorphism in BW gain is well established and attributed to eating disorders, adipose tissue function, and fat distribution in distinct adipose depots [58,59,60,61]. We observed that after performing the time-of-day analysis by the Cosinor approach, food intake in the *BAI3^−/−^* male mice was significantly reduced compared to that in the *BAI3^+/+^* mice during the active night-time feeding phase in mice (Figure 5A, Table 2), potentially contributing to the reduced BW in *BAI3^−/−^* male mice. The hypothalamus is a key regulator of hunger and satiety in response to insulin and leptin signaling. Inhibition of NPY/AgRP neurons and stimulation of POMC/CART neurons decrease food intake, reducing BW [62,63]. Thus, the loss of BAI3 directly or indirectly may modulate NPY/AgRP and POMC/CART functions to regulate BW in adult males.

We observed increased EE in response to the deletion of BAI3 in both male and female mice without changes in total activity and lean mass/BW ratio. Moreover, no difference in EE was achieved at thermoneutrality upon BAI3 deletion in mice, implicating the role of BAT tissue in mediating the effects of BAI3 gene deletion in adaptive thermogenesis. A potential mechanism involves increased in mitochondrial oxidation of FFAs in the BAT to generate heat rather than ATP synthesis [14,64]. We observed reduced WAT tissue weight in both *BAI3^−/−^* male and female mice (Figure 3 and Figure 4). Thus, FFAs generated from the WAT could be utilized as fuels in BAT to enhance EE. The BAT tissue from *BAI3^−/−^* mice exhibited enhanced expression of thermogenic genes (*Dio2, Elov3*, *Ucp1, Pgc1α*), which are known to increase EE by promoting BAT activity [65,66]. Our characterization of *BAI3^−/−^* mice reveals that the loss of BAI3 increases EE by altering BAT activity for adaptive thermogenesis. Additional studies will be required to elucidate the mechanism of BAI3 in regulating energy metabolism in the obesity cold challenge. The outcomes presented in this study implicate that BAI3 is a metabolic regulator whose function can be altered to regulate whole-body energy expenditure, potentially alleviating obesity and metabolic disorders. In conclusion, our findings reveal that BAI3 regulates BW in male and female mice, particularly by regulating energy expenditure via genes involved in adaptive thermogenesis.

## Figures and Tables

**Table 1:** QPCR primer list.

Cyclophilin F	CAG ACG CCA CTG TCG CTT T
Cyclophilin R	TGT CTT TGG AAC TTT GTC TG
Ucp1 F	AGG CTT CCA GTA CCA TTA GGT
Ucp1R	CTG AGT GAG GCA AAG CTG ATT T
Dio2 F	GGC TGA CTT CCT GTT GGT AT
Dio2 R	TTG GTT CCG GTG CTT CTT
Elov3 F	TAC ATC TGG AGG CAG GAG AA
Elov3 R	GGT GGA AGA AGT GAG CGA ATA G
Prdm16 F	CAC AAG TCC TAC ACG CAG TT
Prdm16 R	TTG TTG AGG GAG GAG GTA GT
Pparα F	CGA AGA CAA AGA GGC AGA GG
Pparα R	TGA TGT CAC AGA ACG GCT TC
Pgc1α F	ATG TGT CGC CTT CTT GCT CT
Pgc1α R	ATC TAC TGC CTG GGG ACC TT
Leptin F	GTG GCT TTG GTC CTA TCT GTC
Leptin R	CGT GTG TGA AAT GTC ATT GAT CC
Pparγ1 F	GCG GCT GAG AAA TCA CGT TC
Pparγ1 R	GAA TAT CAG TGG TTC ACC GCT TC
Acox1 F	GTC TCC GTC ATG AAT CCC GA
Acox1 R	TGC GAT GCC AAA TTC CCT CA

**Table 2:** Cosinor analysis attributes for the whole-body energy balance parameters for male *BAI3^+/+^* and *BAI3^−/−^* mice.

Male	Mesor			Amplitude			Phase		
	*BAI3^+/+^*	*BAI3^−/−^*	*p*-Value	*BAI3^+/+^*	*BAI3^−/−^*	*p*-Value	*BAI3^+/+^*	*BAI3^−/−^*	*p*-Value
RER	0.876 ± 0.001	0.883 ± 0.002	<0.01	0.072 ± 0.002	0.083 ± 0.002	<0.01	17.24 ± 0.084	16.73 ± 0.099	<0.01
Energy expenditure	0.0183 ± 0.00015	0.0225 ± 0.00016	6.45 × 10^−19^	0.003458 ± 0.00021	0.004 ± 0.00022	0.07	17.89 ± 0.236	17.58 ± 0.213	0.33
Food intake	0.054785 ± 0.002	0.043344 ± 0.0014	7.57 × 10^−5^	0.032303 ± 0.0028	0.027483 ± 0.0019	0.18	17.22 ± 0.33	16.17 ± 0.27	0.03
Activity	527.513 ± 24.02	460.992 ± 19.14	0.04	392.07 ± 3 4	406.235 ± 27.09	0.74	17.22 ± 0.33	17.76 ± 0.25	0.212

**Table 3:** Cosinor analysis attributes for the whole-body energy balance parameters for female *BAI3^+/+^* and *BAI3^−/−^* mice.

Female	Mesor			Amplitude			Phase		
	BAI3^+/+^	BAI3^−/−^	*p*-Value	BAI3^+/+^	BAI3^−/−^	*p*-Value	BAI3^+/+^	BAI3^−/−^	*p*-Value
RER	0.897 ± 0.002	0.88 ± 0.002	1.05 × 10^−6^	0.056 ± 0.003	0.068 ± 0.002	<0.01	15.52 ± 0.17	16.83 ± 0.13	4.4 × 10^−7^
Energy expenditure	0.0228 ± 0.00015	0.0262 ± 0.00017	2.65 × 10^−15^	0.004022 ± 0.00021	0.004706 ± 0.00025	0.05	17.35 ± 0.20	17.80 ± 0.20	0.13
Food intake	0.058294 ± 0.0022	0.053863 ± 0.0013	0.07	0.025098 ± 0.0031	0.032829 ± 0.0019	0.03	15.70 ± 0.47	16.31 ± 0.22	0.20
Activity	480.247 ± 20	503.837 ± 15	0.34	366.279 ± 29	417.875 ± 21	0.14	18.18 ± 0.30	17.78 ± 0.19	0.24

## Data Availability

The data supporting this study’s findings are available in this article’s methods and/or Supplementary Material. The raw data supporting the conclusions of this article will be made available by the authors without undue reservation.

## Supplementary Materials

The following supporting information can be downloaded at: https://www.mdpi.com/article/10.3390/metabo13060711/s1, Figure S1. Determine the BAI3 mRNA abundance in tissues obtained from *BAI3^+/+^* and *BAI3^−/−^* male mice. The total mRNA was prepared from tissues, and steady-state mRNA abundance of BAI3 expressed relative to the β-actin was determined using qPCR (A–G). Data are reported as the mean ± SEMs, n = 4, * *p* < 0.05. Figure S2. Correlation between lean or fat mass with BW in *BAI3^+/+^* and *BAI3^−/−^* male and female mice. Correlation of lean mass with BW (A and D). Correlation of fat mass with BW (B and E). Lean and fat mass as a percent of BW (C and F).
