# Supplementary material for: Loss of Brain Angiogenesis Inhibitor-3 (BAI3) G-Protein Coupled Receptor in Mice Regulates Adaptive Thermogenesis by Enhancing Energy Expenditure
Source: Metabolites. 2023 May 31;13(6):711. doi: 10.3390/metabo13060711 (PMC10301052; doi:10.3390/metabo13060711)
Supplement: Supplementary file 1 [file metabolites-13-00711-s001.zip › metabolites-2316799-supplementary.pdf]

Supplementary Data

Loss of Brain Angiogenesis Inhibitor-3 (BAI3) G-Protein Coupled Receptor in Mice Regulates Adaptive Thermogenesis by Enhancing Energy Expenditure

Haifa Alsharif [1,2], Mary N. Latimer [3], Katherine C. Perez [1,2], Justin Alexander [1,2], Md Mostafizur Rahman [1,2], Anil K. Challa [5], Jeong-A. Kim [1,2], Sasanka Ramanadham [2,4], Martin Young [3] and Sushant Bhatnagar [1,2,*]

[1] Department of Medicine, Division of Endocrinology, Diabetes, and Metabolism, Heersink School of Medicine, University of Alabama at Birmingham, Birmingham, AL 35294, USA; saba2323@uab.edu (H.A.); justinalexander@uabmc.edu (J.A.); jeongakim@uabmc.edu (J.-A.K.); kperez24@uab.edu (K.C.P.); mostafizurrahman@uabmc.edu (M.M.R.)

[2] Comprehensive Diabetes Center, University of Alabama at Birmingham, Birmingham, AL 35294, USA

[3] Division of Cardiovascular Disease, Department of Medicine, Heersink School of Medicine, University of Alabama at Birmingham, Birmingham, AL 35294, USA; martinyoung@uabmc.edu (M.Y.)

[4] Department of Biology, University of Alabama at Birmingham, Birmingham, AL 35294, USA; akchalla@uab.edu

[5] Department of Cell, Developmental, and Integrative Biology, University of Alabama at Birmingham, Birmingham, AL 35294, USA

* Correspondence: sushantbhatnagar@uabmc.edu; Tel.: +205-996-0164; Fax: +205-996-5220

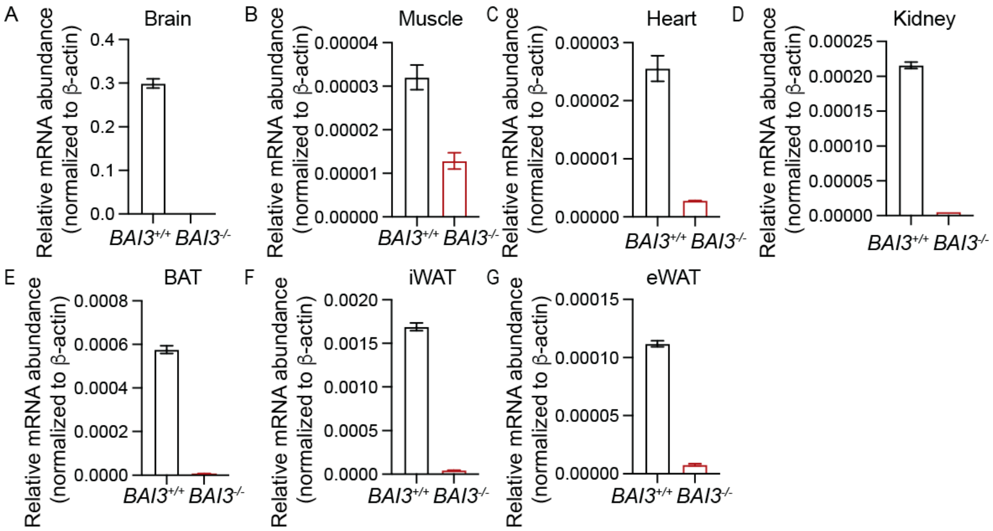

Figure S1. Determine the BAI3 mRNA abundance in tissues obtained from $BAI3^{+/+}$ and $BAI3^{-/-}$ male mice. The total mRNA was prepared from tissues, and steady-state mRNA abundance of BAI3 expressed relative to the β-actin was determined using qPCR **(A-G).** Data are reported as the mean ± SEMs, n=4, * $P < 0.05$.

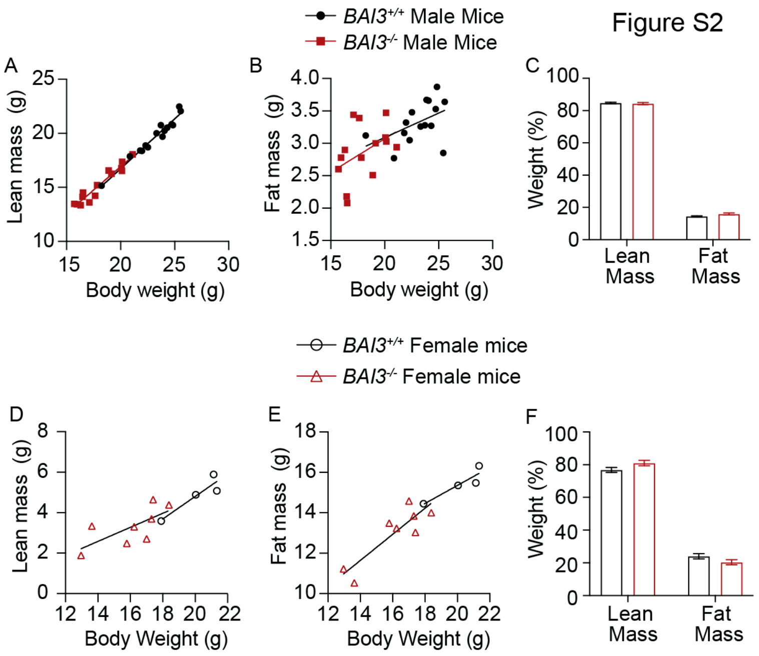

Figure S2. Correlation between lean or fat mass with BW in *BAI3*$^{+/+}$ and *BAI3*$^{-/-}$ male and female mice. Correlation of lean mass with BW **(A and D)**. Correlation of fat mass with BW (**B and E**). Lean and fat mass as a percent of BW (**C and F**).
